# Supplementary material for: Pyruvate Dehydrogenase Kinase 1 inhibition mediated oxidative phosphorylation enhancement in cartilage promotes osteoarthritis progression
Source: BMC Musculoskelet Disord. 2023 Jul 20;24:597. doi: 10.1186/s12891-023-06585-6 (PMC10357736; doi:10.1186/s12891-023-06585-6)
Supplement: Supplementary file 1 — Additional file 1. [file 12891_2023_6585_MOESM1_ESM.zip › MF.Gsea.1653627316242/GOMF_VITAMIN_B6_BINDING.html]

Details for gene set GOMF\_VITAMIN\_B6\_BINDING[GSEA]

|  || Dataset | 55457\_collapsed\_to\_symbols.cls.cls #OA\_versus\_normal.cls.cls #OA\_versus\_normal\_repos |
| Phenotype | cls.cls#OA\_versus\_normal\_repos |
| Upregulated in class | OA |
| GeneSet | GOMF\_VITAMIN\_B6\_BINDING |
| Enrichment Score (ES) | 0.32801113 |
| Normalized Enrichment Score (NES) | 1.291667 |
| Nominal p-value | 0.112299465 |
| FDR q-value | 0.4053433 |
| FWER p-Value | 1.0 |
Table: GSEA Results Summary

  

Fig 1: Enrichment plot: GOMF\_VITAMIN\_B6\_BINDING      
 Profile of the Running ES Score & Positions of GeneSet Members on the Rank Ordered List

  

| SYMBOL | TITLE | RANK IN GENE LIST | RANK METRIC SCORE | RUNNING ES | CORE ENRICHMENT || 1 | GOT1 | glutamic-oxaloacetic transaminase 1 [Source:HGNC Symbol;Acc:HGNC:4432] | 59 | 0.974 | 0.0742 | Yes |
| 2 | ALAS1 | 5'-aminolevulinate synthase 1 [Source:HGNC Symbol;Acc:HGNC:396] | 288 | 0.757 | 0.1176 | Yes |
| 3 | PLPBP | pyridoxal phosphate binding protein [Source:HGNC Symbol;Acc:HGNC:9457] | 637 | 0.610 | 0.1399 | Yes |
| 4 | GCAT | glycine C-acetyltransferase [Source:HGNC Symbol;Acc:HGNC:4188] | 1095 | 0.497 | 0.1445 | Yes |
| 5 | GOT2 | glutamic-oxaloacetic transaminase 2 [Source:HGNC Symbol;Acc:HGNC:4433] | 1308 | 0.457 | 0.1649 | Yes |
| 6 | SHMT2 | serine hydroxymethyltransferase 2 [Source:HGNC Symbol;Acc:HGNC:10852] | 1518 | 0.426 | 0.1831 | Yes |
| 7 | NFS1 | NFS1 cysteine desulfurase [Source:HGNC Symbol;Acc:HGNC:15910] | 1741 | 0.393 | 0.1976 | Yes |
| 8 | PDXK | pyridoxal kinase [Source:HGNC Symbol;Acc:HGNC:8819] | 1923 | 0.370 | 0.2134 | Yes |
| 9 | PYGM | "glycogen phosphorylase, muscle associated [Source:HGNC Symbol;Acc:HGNC:9726]" | 1952 | 0.367 | 0.2409 | Yes |
| 10 | TAT | tyrosine aminotransferase [Source:HGNC Symbol;Acc:HGNC:11573] | 1979 | 0.363 | 0.2682 | Yes |
| 11 | OAT | ornithine aminotransferase [Source:HGNC Symbol;Acc:HGNC:8091] | 2074 | 0.353 | 0.2895 | Yes |
| 12 | PYGB | glycogen phosphorylase B [Source:HGNC Symbol;Acc:HGNC:9723] | 2400 | 0.315 | 0.2896 | Yes |
| 13 | KYNU | kynureninase [Source:HGNC Symbol;Acc:HGNC:6469] | 2502 | 0.303 | 0.3062 | Yes |
| 14 | GAD1 | glutamate decarboxylase 1 [Source:HGNC Symbol;Acc:HGNC:4092] | 2534 | 0.299 | 0.3280 | Yes |
| 15 | SPTLC1 | serine palmitoyltransferase long chain base subunit 1 [Source:HGNC Symbol;Acc:HGNC:11277] | 2913 | 0.259 | 0.3196 | No |
| 16 | SGPL1 | sphingosine-1-phosphate lyase 1 [Source:HGNC Symbol;Acc:HGNC:10817] | 3462 | 0.206 | 0.2936 | No |
| 17 | PYGL | glycogen phosphorylase L [Source:HGNC Symbol;Acc:HGNC:9725] | 3826 | 0.174 | 0.2794 | No |
| 18 | GLDC | glycine decarboxylase [Source:HGNC Symbol;Acc:HGNC:4313] | 3913 | 0.163 | 0.2859 | No |
| 19 | KYAT3 | kynurenine aminotransferase 3 [Source:HGNC Symbol;Acc:HGNC:33238] | 4128 | 0.143 | 0.2808 | No |
| 20 | SRR | serine racemase [Source:HGNC Symbol;Acc:HGNC:14398] | 4397 | 0.121 | 0.2698 | No |
| 21 | SDS | serine dehydratase [Source:HGNC Symbol;Acc:HGNC:10691] | 4579 | 0.106 | 0.2643 | No |
| 22 | CBS | cystathionine beta-synthase [Source:HGNC Symbol;Acc:HGNC:1550] | 5285 | 0.049 | 0.2134 | No |
| 23 | MTARC2 | mitochondrial amidoxime reducing component 2 [Source:HGNC Symbol;Acc:HGNC:26064] | 6076 | -0.010 | 0.1528 | No |
| 24 | THNSL2 | threonine synthase like 2 [Source:HGNC Symbol;Acc:HGNC:25602] | 6395 | -0.034 | 0.1308 | No |
| 25 | PSAT1 | phosphoserine aminotransferase 1 [Source:HGNC Symbol;Acc:HGNC:19129] | 6585 | -0.049 | 0.1201 | No |
| 26 | HDC | histidine decarboxylase [Source:HGNC Symbol;Acc:HGNC:4855] | 6599 | -0.050 | 0.1231 | No |
| 27 | ABAT | 4-aminobutyrate aminotransferase [Source:HGNC Symbol;Acc:HGNC:23] | 6804 | -0.063 | 0.1123 | No |
| 28 | ETNPPL | ethanolamine-phosphate phospho-lyase [Source:HGNC Symbol;Acc:HGNC:14404] | 6996 | -0.079 | 0.1039 | No |
| 29 | SCLY | selenocysteine lyase [Source:HGNC Symbol;Acc:HGNC:18161] | 7044 | -0.083 | 0.1069 | No |
| 30 | PDXDC1 | pyridoxal dependent decarboxylase domain containing 1 [Source:HGNC Symbol;Acc:HGNC:28995] | 7373 | -0.108 | 0.0901 | No |
| 31 | MTARC1 | mitochondrial amidoxime reducing component 1 [Source:HGNC Symbol;Acc:HGNC:26189] | 7500 | -0.118 | 0.0899 | No |
| 32 | ALB | albumin [Source:HGNC Symbol;Acc:HGNC:399] | 7684 | -0.133 | 0.0864 | No |
| 33 | AGXT | alanine--glyoxylate and serine--pyruvate aminotransferase [Source:HGNC Symbol;Acc:HGNC:341] | 8115 | -0.164 | 0.0662 | No |
| 34 | GPT | glutamic--pyruvic transaminase [Source:HGNC Symbol;Acc:HGNC:4552] | 8411 | -0.186 | 0.0583 | No |
| 35 | DDC | dopa decarboxylase [Source:HGNC Symbol;Acc:HGNC:2719] | 8605 | -0.202 | 0.0596 | No |
| 36 | KYAT1 | kynurenine aminotransferase 1 [Source:HGNC Symbol;Acc:HGNC:1564] | 8633 | -0.203 | 0.0739 | No |
| 37 | MOCOS | molybdenum cofactor sulfurase [Source:HGNC Symbol;Acc:HGNC:18234] | 8721 | -0.210 | 0.0842 | No |
| 38 | GAD2 | glutamate decarboxylase 2 [Source:HGNC Symbol;Acc:HGNC:4093] | 9249 | -0.252 | 0.0636 | No |
| 39 | SPTLC2 | serine palmitoyltransferase long chain base subunit 2 [Source:HGNC Symbol;Acc:HGNC:11278] | 9534 | -0.277 | 0.0639 | No |
| 40 | SHMT1 | serine hydroxymethyltransferase 1 [Source:HGNC Symbol;Acc:HGNC:10850] | 9727 | -0.294 | 0.0727 | No |
| 41 | PNPO | pyridoxamine 5'-phosphate oxidase [Source:HGNC Symbol;Acc:HGNC:30260] | 10239 | -0.345 | 0.0609 | No |
| 42 | ALAS2 | 5'-aminolevulinate synthase 2 [Source:HGNC Symbol;Acc:HGNC:397] | 10247 | -0.346 | 0.0883 | No |
| 43 | CTH | cystathionine gamma-lyase [Source:HGNC Symbol;Acc:HGNC:2501] | 11108 | -0.444 | 0.0573 | No |
| 44 | SPTLC3 | serine palmitoyltransferase long chain base subunit 3 [Source:HGNC Symbol;Acc:HGNC:16253] | 11416 | -0.489 | 0.0729 | No |
| 45 | CSAD | cysteine sulfinic acid decarboxylase [Source:HGNC Symbol;Acc:HGNC:18966] | 11649 | -0.525 | 0.0973 | No |
Table: GSEA details [plain text format]

  

Fig 2: GOMF\_VITAMIN\_B6\_BINDING      
 Blue-Pink O' Gram in the Space of the Analyzed GeneSet

  

Fig 3: GOMF\_VITAMIN\_B6\_BINDING: Random ES distribution      
 Gene set null distribution of ES for **GOMF\_VITAMIN\_B6\_BINDING**
